# Performance of wearable finger ring trackers for diagnostic sleep measurement in the clinical context

**DOI:** 10.1038/s41598-025-93774-z

**Published:** 2025-03-19

**Authors:** Sebastian Herberger, Christoph Aurnhammer, Sophie Bauerfeind, Tomas Bothe, Thomas Penzel, Ingo Fietze

**Affiliations:** 1 Interdisciplinary Center of Sleep Medicine, Charité University Medicine Berlin, Berlin, Germany; 2 Mentalab GmbH, Munich, Germany; 3 Center for Space Medicine and Extreme Environments, Charité University Medicine Berlin, Berlin, Germany

**Keywords:** Diagnostic markers, Translational research

## Abstract

Ring-trackers are a growing consumer wearable category that provide a number of sleep metrics, yet their measurement accuracy remains poorly understood. Previous validation studies have mainly focused on healthy individuals, while a significant part of the potential present and future value lies in applications on non-healthy subjects. To enable applications in research and medical applications, rigorous evaluation of performance in clinical settings against the gold-standard polysomnography is needed. To address this knowledge gap, we investigated how the measurements of three commercially available ring trackers (Oura, SleepOn, Circul) perform against polysomnography in a university sleep lab population with a diverse set sleep-related disorders as well as sleep-unrelated medical conditions. We evaluated individual-level and group-level discrepancies of standard sleep measures and conducted an epoch-by-epoch analysis of sleep staging classification performance using a standardized analysis framework. While average group-level sleep measures are similar (e.g., TST differences between rings and gold standard were below 12 min for the Oura ring), individual-level differences often remained large. Ring-derived sleep metrics were characterized by complex bias, indicating that their correction is non-trivial. Sleep/Wake distinction of the Oura and SleepOn rings reached similar performance as previously reported for healthy individuals (~ 85% accuracy), but was worse for the Circul ring (~ 65% accuracy). Sleep stage classification (Wake, Light, Deep, REM sleep) sensitivity ranged from 0.14 (REM sleep classification of the Circul ring) to 0.58 (Light sleep classification of the SleepOn ring). Across all sleep stages, the Oura and SleepOn ring performed similarly (53.18% and 50.48% accuracy), whereas the Circul ring performed worse (35.06% accuracy). Our findings confirm recent descriptions of device-related bias and additionally uncover practical limitations in the application in a real-world sleep laboratory patient cohort. Critically, while some devices may demonstrate reasonable agreement with PSG *on average*, this agreement masks substantial individual-level inaccuracies, prohibiting their use in clinical sleep medicine, as accurate assessment of individual nights, including both nights with exceptionally low or high sleep quality and quantity, is essential for patient care.

**Supplementary Information:**

The online version contains supplementary material available at 10.1038/s41598-025-93774-z.

## Introduction

Personal health and consumer sleep-tracking technology devices (CSTs) are seeing unprecedented, widespread adoption in consumer markets worldwide^1^. The rather recent category of finger ring trackers represents a novel type that holds potential as a low-cost and easily accessible medical diagnostic and research tool^2^. The clinical gold standard for sleep measurement, polysomnography (PSG), involves the simultaneous, supervised recording of multiple biosignals in a sleep lab, which requires significant resources. Compared to CSTs, PSG is in limited supply and often associated with long wait times^3–5^. Thus, CST devices, such as ring trackers could significantly lower the barrier for sleep investigations and sleep tracking, allowing for longitudinal sleep measurements at unprecedented scale in natural at-home environments^6^. Most CST devices provide outputs that resemble the measures of PSG, such as per-epoch sleep stage classifications or blood oxygen level-derived analyses, thereby suggesting that they might indicate true and accurate measurements. However, large knowledge gaps remain regarding the measurement accuracy of CST. While the promise of cheaper and easier alternatives to traditional PSG is intriguing, rigorous scientific evaluation of their measurement accuracy compared to the clinical gold standard is needed to validate CSTs for clinical or research purposes.

Actigraphy is an established wearable sleep measurement technology that can be considered a comparable precursor to CSTs^7^. Actigraphs are equipped with an accelerometer sensor and are worn on the wrist, bringing with them many of the same benefits of CSTs. In validation studies against PSG, actigraphy has reached a sensitivity range of 72–97%, but a specificity of only 28–67% in detecting sleep, highlighting the difficulty of accurately identifying wake periods^8–14^. Ring tracking devices have been subject to scientific evaluation over recent years, however, studies have focused on samples of healthy, and, typically, young individuals. In these studies examining the ability of ring trackers to discriminate between wake and sleep, sleep detection sensitivity was ≥ 0.89, whereas specificity ranged from 0.18 to 0.54^15–19^, mirroring the same problem in detecting wake periods. The classification of individual sleep stages has been even less accurate, with sensitivity ranging from 0.28 to 0.82 and specificity ranging from 0.18 to 0.95 across different sleep stages^16–19^. Furthermore, discrepancy analyses of standard sleep measures such as total sleep time (TST), sleep-onset latency (SOL), sleep efficiency (SE) and wake-after-sleep-onset (WASO) often revealed systematic bias in CSTs compared to PSG, indicating that low values of TST and SE are underestimated by many devices, whereas higher values of SOL and WASO are often characterized by large variability in device accuracy, yielding both over- and underestimation^16–19^.

Quantitative sleep measures (TST, SOL, SE, WASO) derived from sleep/wake discrimination are key measures of sleep quantity and quality^20,21^, and are critical in the assessment of sleep disorders. For instance, insomnia is defined as a wake phase of more than 30 min per night, occurring at least three times per week, combined with daytime symptoms (ICD-10-CM). Professional use of CST’s requires measurement performance that is close enough to PSG, as well as validation in a sample of real-world patients, including typical patient phenotypes with sleep-related pathologies and comorbidities, ideally in the context of clinical routine work. Prior research of clinical applications has investigated the diagnostic potential of ring trackers for specific disorders, such as obstructive sleep apnoea (OSA)^22,23^, and there is a developing landscape of consumer trackers for home-sleep testing of sleep disordered breathing (such as the Samsung Galaxy Watch and Apple Watch apnea detection features^24^), all of which are predominantly focused on blood oxygen level. In comparison to blood oxygen-level related diagnostics, the accurate classification of sleep state and stages in clinical populations is more complex and remains mostly uncharted territory.

Recent studies comparing sleep trackers to gold-standard PSG in healthy individuals highlighted methodological shortcomings present in a large body of previous literature^25^. For instance, previous studies often tested only for *constant bias* which could in principle be subtracted from CST measures (*calibration index*) to correct the systematic measurement error. Menghini and colleagues^25^ have proposed a standardized framework designed to facilitate thorough evaluation of sleep tracking devices against a gold-standard reference while testing statistical assumptions and adapting the estimation of device bias where needed as well as to improve comparability across sleep tracking technology evaluation studies. Recent studies of ring trackers are starting to offer a more thorough characterization of the differences between ring tracker devices and PSG, revealing that in many cases constant bias is inadequate to correct CST^16–18,25^. The present study applies the standard measurement performance evaluation framework by Menghini and colleagues to a sample of all-comers from a university sleep lab patient population with a diverse set of previous sleep-related (e.g., various sleep apnoea syndromes, restless-legs-syndrome, insomnia, hypersomnia, hypercapnia, narcolepsy) and sleep-unrelated disorders to investigate the performance of ring trackers in a real-world setting, with their potential use as diagnostic and research tools in mind. Recordings were taken from three ring trackers (Oura^26^, SleepOn^27^, Circul^28^) and PSG, with the goal of a direct head-to-head comparison of the CSTs against a gold-standard reference, focusing both on standard sleep measures as well as epoch-by-epoch sleep stage classification.

## Results

### Device data acquisition

In a total of 45 measurement nights on 45 different patients, data was successfully acquired for 31 nights (Oura, 69%), 35 nights (SleepOn, 78%) and 31 nights (Circul, 69%). Unusable measurements (dropouts) resulted from human and machine error. Dropouts occurred due to ring recording error, PSG recording error, protocol error, and problems with ring fit, such as rings that fell off or were taken off prematurely due to poor fit (see Suppl. Table 5 for a detailed overview). All subsequent analyses use the maximum available data for each sleep tracker ring. As a consequence of this, the results reported for each ring are based on different subsets of participants (Tables 1, 2, 3 and 4).

**Table 1:** Demographic data for included subjects for each ring. TIB is calculated based on complete observations from both ring tracker and polysomnography.

Ring	Measurements	Age	Gender (% female)	Height	Weight	BMI	TIB (complete data)
Oura	31	53.60 (12.90)	41.90	174.80 (11.20)	100.70 (23.40)	33.10 (7.90)	437.53 (62.75)
SleepOn	35	54.50 (12.30)	45.70	175.00 (11.50)	102.00 (22.70)	33.50 (7.70)	440.72 (65.26)
Circul	31	56.10 (9.80)	51.60	173.80 (9.90)	99.00 (20.50)	32.90 (7.00)	438.85 (70.35)

**Table 2:** Differences between ring tracker sleep metrics and PSG sleep metrics in the format *mean (SD; range)*.

	Oura			SleepOn			Circul		
Metric	Device mean (SD)	Reference Mean (SD)	Difference (SD; range)	Device mean (SD)	Reference Mean (SD)	Difference (SD; range)	Device mean (SD)	Reference Mean (SD)	Difference (SD; range)
TST (mins.)	379.11 (70.49)	367.37 (74.67)	11.74 (37.11; −54–97.5)	418.11 (61.77)	367.66 (77.54)	50.46 (37.42; −4–146)	337.73 (88.45)	369.18 (75.69)	−31.45 (70.06; −167–111.5)
SE (%)	86.42 (7.65)	83.4 (9.55)	3.02 (9.78; −13.94–30.73)	89.38 (7.63)	78.14 (12.28)	11.24 (9.17; −0.86–37.41)	71.66 (12.14)	78.51 (9.67)	−6.85 (15.79; −39.24–24.05)
SOL (mins.)	6.44 (7.42)	9.61 (8.51)	−3.18 (12.52; −34.5–32.5)	16.33 (24.04)	35.44 (32.6)	−19.11 (23.56; −88–7)	7.29 (25.93)	32.53 (28.69)	−25.24 (17.82; −83–0)
WASO (mins.)	51.98 (29.94)	60.55 (34.97)	−8.56 (33.02; −90.5–66.5)	34.59 (28.89)	65.93 (39.13)	−31.34 (32.19; −116–46.5)	122.89 (59.02)	66.19 (34.5)	56.69 (61.62; −32–173)
Light (mins.)	200.16 (69.85)	214 (47.27)	−13.84 (79.43; −162–234.5)	233.63 (78)	217.64 (63.92)	15.99 (67.67; −112.5–166)	204.35 (70.15)	222.9 (64.04)	−18.55 (63.92; −153.5–136.5)
Deep (mins.)	79.76 (56.59)	85.74 (48.35)	−5.98 (67.79; −211.5–141.5)	114.97 (75.93)	83.19 (48.35)	31.79 (61.33; −80–172.5)	64.35 (34.38)	79.48 (51.28)	−15.13 (50.25; −122–63)
REM (mins.)	99.19 (51.5)	67.63 (31.26)	31.56 (44.04; −40–172)	69.51 (61.98)	66.83 (37.52)	2.69 (57.4; −67.5–153)	69.02 (20.06)	66.79 (37.36)	2.23 (37.86; −111–67.5)

Descriptive statistics are computed across subjects.

**Table 3:** Sleep/Wake classification accuracy of the ring trackers compared to polysomnography.

	Oura	SleepOn	Circul
Accuracy	85.03 (7.72)	84.00 (8.84)	65.66 (12.03)
Sleep Sensitivity (= Wake Specificity)	92.78 (6.09)	96.84 (2.76)	73.92 (12.98)
Wake Sensitivity (= Sleep Specificity)	46.19 (18.67)	37.40 (18.95)	35.43 (24.83)
Kappa	0.43	0.44	0.10

**Table 4:** Epoch-by-epoch group-level proportional error matrix on the sample data. PSG-based N1 + N2 is labeled “light”; PSG-based N3 is labeled “deep”; REM is rapid eye movement sleep.

		Reference			
		Wake	Light	Deep	REM
Oura	Wake	0.46 (0.19) [0.40, 0.53]	0.1 (0.1) [0.06, 0.13]	0.01 (0.02) [0.00, 0.01]	0.06 (0.09) [0.03, 0.09]
	Light	0.38 (0.15) [0.33, 0.43]	0.56 (0.17) [0.51, 0.62]	0.33 (0.27) [0.23, 0.42]	0.30 (0.24) [0.22, 0.38]
	Deep	0.07 (0.08) [0.04, 0.09]	0.12 (0.13) [0.08, 0.17]	0.50 (0.32) [0.39, 0.62]	0.08 (0.12) [0.04, 0.12]
	REM	0.09 (0.12) [0.04, 0.12]	0.21 (0.13) [0.16, 0.25]	0.16 (0.25) [0.07, 0.24]	0.55 (0.29) [0.45, 0.65]
SleepOn	Wake	0.37 (0.19) [0.31, 0.43]	0.05 (0.04) [0.03, 0.06]	0.01 (0.03) [0.00, 0.02]	0.01 (0.02) [0.00, 0.01]
	Light	0.50 (0.17) [0.45, 0.56]	0.58 (0.16) [0.53, 0.64]	0.32 (0.25) [0.24, 0.40]	0.43 (0.28) [0.33, 0.52]
	Deep	0.06 (0.09) [0.03, 0.08]	0.21 (0.16) [0.16, 0.27]	0.48 (0.31) [0.38, 0.58]	0.31 (0.25) [0.22, 0.39]
	REM	0.06 (0.11) [0.02, 0.09]	0.15 (0.16) [0.10, 0.20]	0.19 (0.25) [0.10, 0.27]	0.26 (0.25) [0.17, 0.33]
Circul	Wake	0.35 (0.25) [0.27, 0.44]	0.29 (0.17) [0.23, 0.34]	0.17 (0.14) [0.11, 0.22]	0.30 (0.26) [0.21, 0.39]
	Light	0.37 (0.20) [0.30, 0.44]	0.45 (0.20) [0.38, 0.51]	0.52 (0.24) [0.44, 0.60]	0.41 (0.24) [0.33, 0.50]
	Deep	0.13 (0.12) [0.09, 0.18]	0.13 (0.09) [0.10, 0.16]	0.17 (0.18) [0.10, 0.22]	0.14 (0.18) [0.07, 0.20]
	REM	0.14 (0.13) [0.09, 0.18]	0.14 (0.06) [0.12, 0.16]	0.14 (0.19) [0.08, 0.20]	0.14 (0.14) [0.09, 0.19]

Results are reported as *mean (standard deviation) [95% confidence intervals]*. Diagonal entries represent sensitivity (bold) and off-diagonal entries represent classification errors.

### Discrepancy analysis

Group-level and individual-level discrepancies revealed a wide range of differences between ring and PSG sleep metrics. On average, ring trackers were closely aligned with PSG for some sleep metrics: For instance, the Oura ring overestimated deep sleep duration by only 5.98 min from PSG on average (SD = 67.79 min.). In other cases, average differences were larger: For example, the SleepOn ring overestimated deep sleep duration by 31.79 min (SD = 61.33 min.). Across individuals, large variability in the accuracy of ring trackers was observed, as indicated by the range of positive and negative difference terms (Table 2). For instance, the light sleep duration estimate from the Circul ring ranged from − 153.5 min underestimation to 136.5 min overestimation around the average difference.

#### Discrepancy analysis

**Fig. 1:**
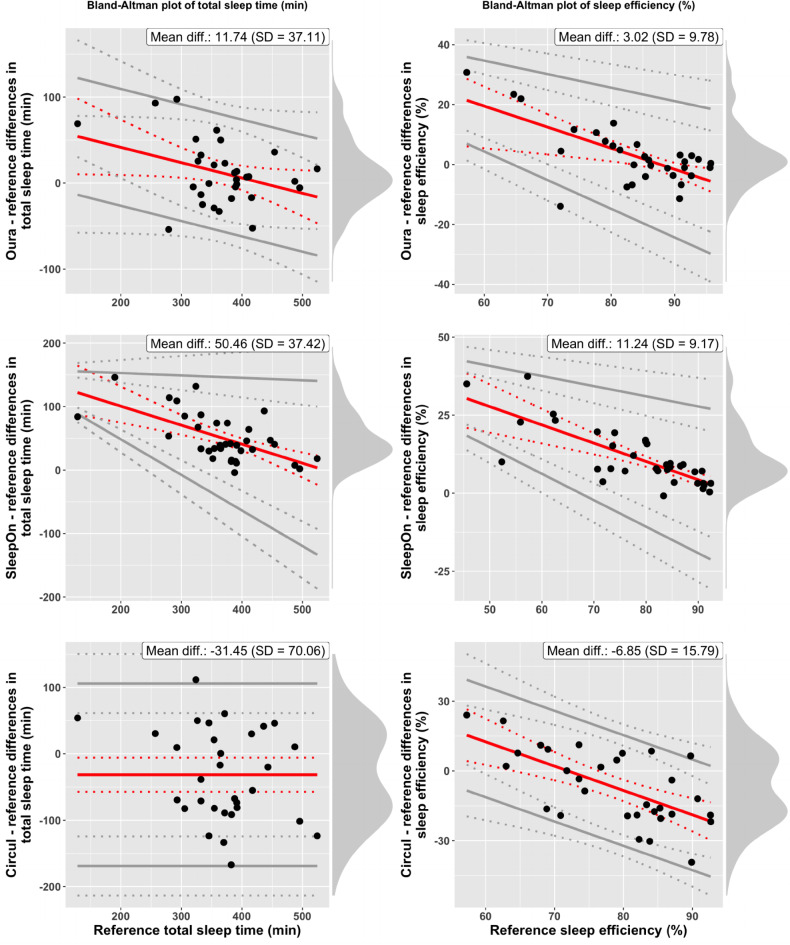
Bland–Altman plots of *total sleep time* (TST, left column) and *sleep efficiency* (SE, right column) for the three sleep tracker devices (row 1: Oura; row 2: SleepOn; row 3 Circul) compared to reference (PSG). X-axis represents gold-standard PSG measures. Red solid lines represent bias, gray solid lines represent the 95% LOAs, both with 95% CIs (dotted lines). Black points represent individual-level observations. Density diagrams on the right side of the plots indicate the distribution of the differences. Plots are adjusted for the specific case of compliance with the assumptions for discrepancy analysis: all fulfilled (TST-Circul), proportional bias and homoscedastic differences (TST-Oura, SE-Circul), both proportional bias and heteroscedastic differences (TST-SleepOn, SE-Oura, SE-SleepOn). Bootstrapped confidence intervals were computed and log-transformation applied where statistical assumptions were not met (TST-SleepOn, SE-Oura, SE-SleepOn).

**Fig. 2:**
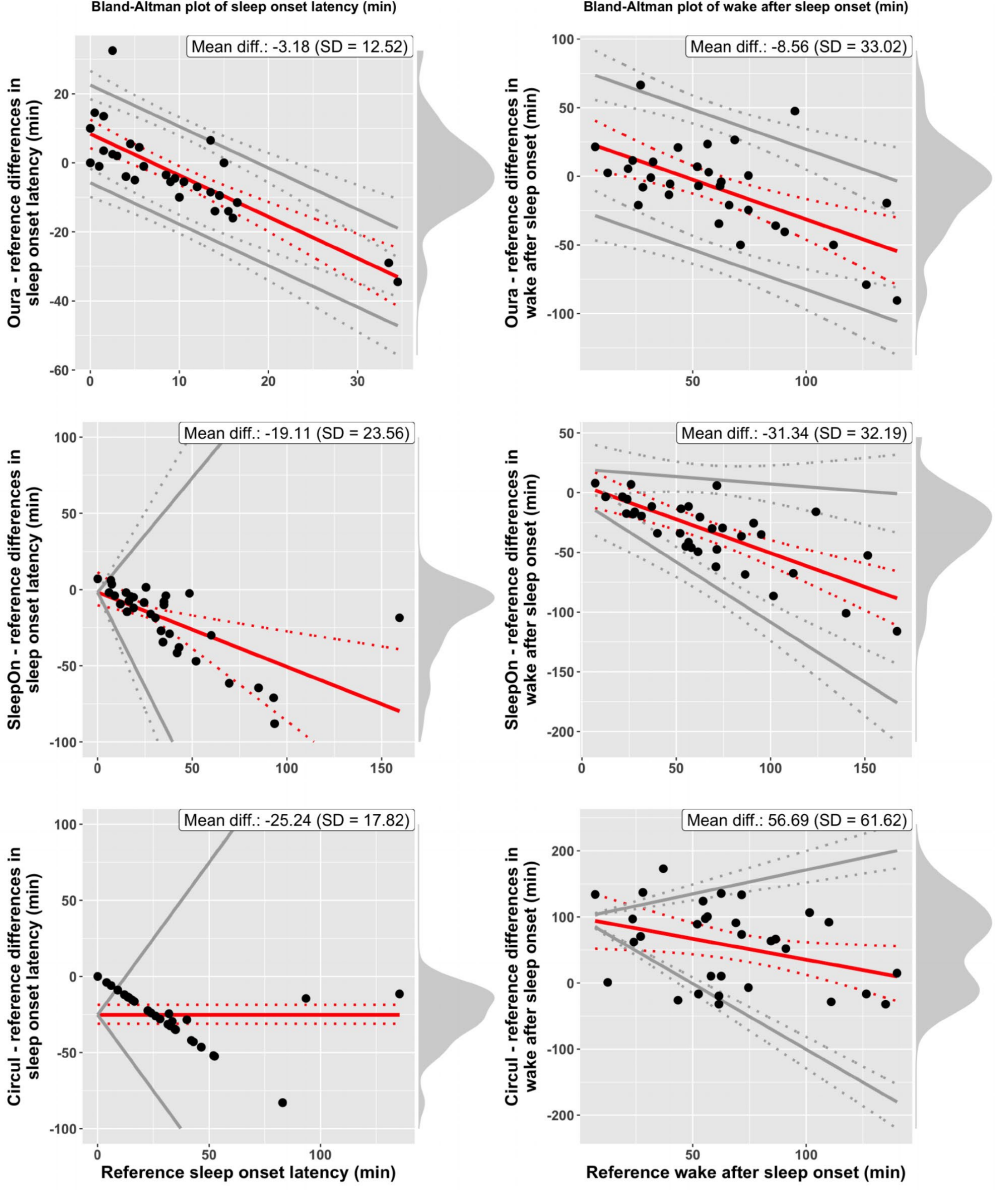
Bland–Altman plots of *sleep onset latency* (SE, left column) and *wake after sleep onset* (WASO, right column) for the three sleep tracker devices (row 1: Oura; row 2: SleepOn; row 3 Circul) compared to reference (PSG). Red solid lines represent bias, gray solid lines represent the 95% LOAs, both with 95% CIs (dotted lines). Black points represent individual-level observations. Density diagrams on the right side of the plots indicate the distribution of the differences. Plots are adjusted for the specific case of compliance with the assumptions for discrepancy analysis: constant bias and homoscedastic differences (SOL-Circul), proportional bias and homoscedastic differences (SOL-Oura, SOL-SleepOn, WASO-Oura, WASO-Circul), proportional bias and heteroscedastic differences (WASO-SleepOn). Bootstrapped confidence intervals were computed and log-transformation applied where statistical assumptions were not met (SOL-SleepOn, SOL-Circul, WASO-Circul).

**Fig. 3:**
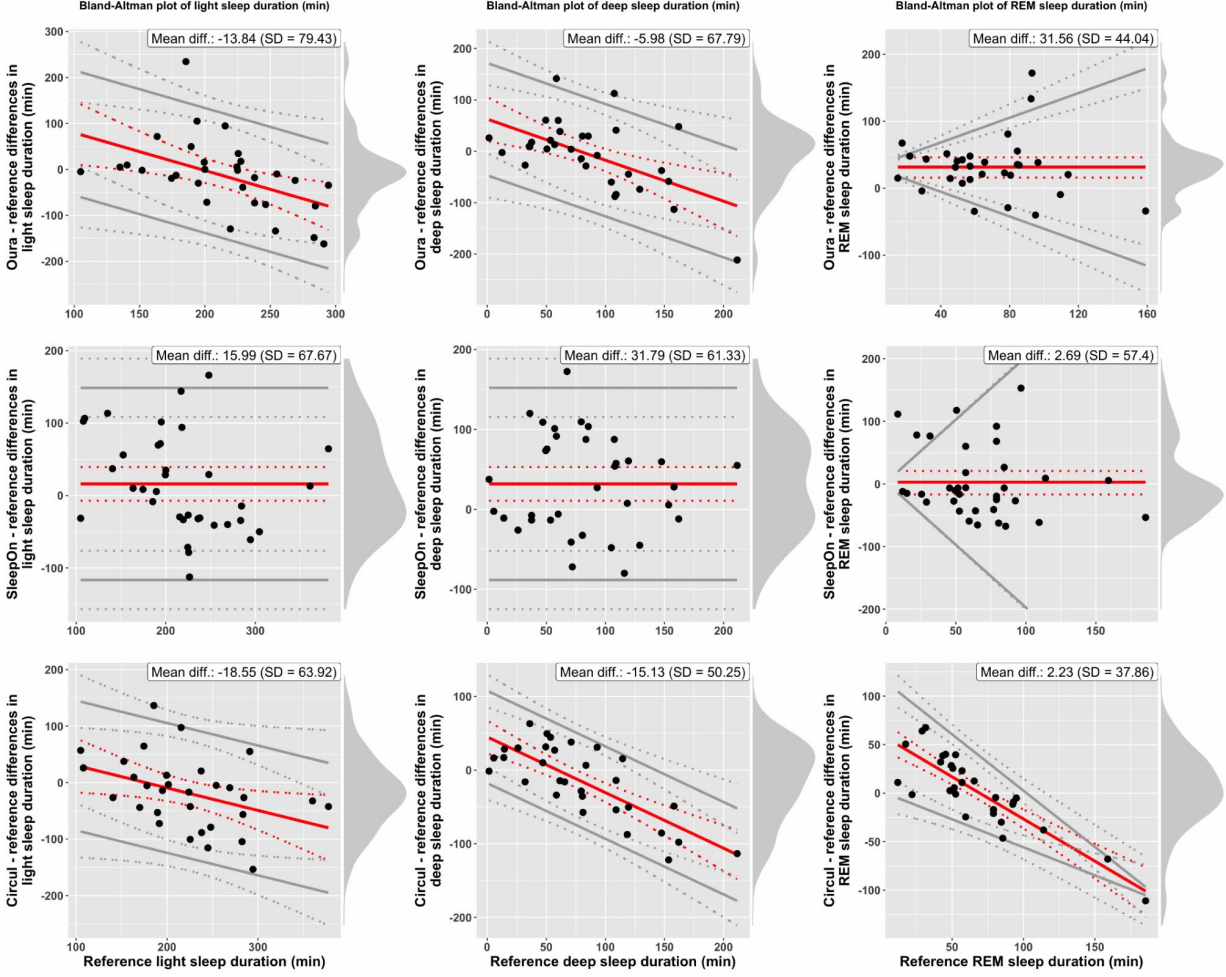
Bland–Altman plots of *light sleep duration* (left column), *deep sleep duration* (mid column), *REM sleep duration* (right column) for the three sleep tracker devices (row 1: Oura; row 2: SleepOn; row 3 Circul) compared to reference (PSG). Red solid lines represent bias, gray solid lines represent the 95% LOAs, both with 95% CIs (dotted lines). Black points represent individual-level observations. Density diagrams on the right side of the plots indicate the distribution of the differences. Plots are adjusted for the specific case of compliance with the assumptions for discrepancy analysis: all fulfilled (Light-SleepOn, Deep-SleepOn), constant bias and homoscedastic differences (REM-SleepOn), constant bias but heteroscedastic differences (REM-Oura), proportional bias but homoscedastic differences (Light-Oura, Light-Circul, Deep-Oura, Deep-Circul), proportional bias and heteroscedastic differences (REM-Circul). Bootstrapped confidence intervals were computed and log-transformation applied where statistical assumptions were not met (REM-Oura, REM-SleepOn).

The analysis of group-level and individual-level differences focuses on computing bias estimates (Suppl. Table 3), the range of differences observed across subjects and on visual inspection of Bland-Altman plots. Horizontal red lines indicate constant bias, whereas inclined red lines indicate proportional bias. Parallel gray lines for limits of agreement indicate homoscedasticity, whereas gray lines with different slopes indicate heteroscedasticity. This is the case if the standard deviation of the differences in device and reference measures varies significantly as a function of the scale of measurement. Note that while the x-axis of the Bland-Altman (BA) plots always represents gold-standard PSG measures, x-axis scales differ across rings, due to data dropouts leading to different subsets of participants entering the analysis of each ring. Figure 1 displays BA plots of TST (left column) and SE (right column) for all three rings (row 1: Oura; row 2: SleepOn; row 3: Circul), compared to reference (PSG). Figure 2 displays BA plots of SOL and WASO and Fig. 3 displays BA plots of light sleep, deep sleep, and REM sleep duration for all three rings.

#### Total sleep time

Compared to TST values computed from PSG, the Oura ring overestimated TST by 11.74 min on average (SD = 37.11 min.), and individual-level differences ranged from − 54.00 min to 97.50 min. The SleepOn ring overestimated TST by 50.46 min on average (SD = 37.42; individual-level difference range from − 4.00 min to 146.00 min), and the Circul ring underestimated TST by 31.45 min on average (SD = 70.06 min.; individual-level difference range from − 167.00 min to 111.50 min). For TST, significant proportional bias was detected for the Oura ring, where lower (true) values of TST were overestimated to a stronger degree. The SleepOn ring was affected by significant proportional bias, leading to significant overestimation of TST for all but very high measures of TST. Differences between TST measures of the Circul ring and reference were homoscedastic, but negative constant bias was present.

#### Sleep efficiency

SE percentages were overestimated by 3.02% on average by the Oura ring (SD = 9.78%), with an error range of -13.04–30.73%. The SleepOn ring overestimated SE by 11.24% (SD = 9.17%; range from − 0.89 to 37.41%) and the Circul ring underestimated SE by 6.85% (SD = 15.79; range from − 39.24 to 24.05%). SE was affected by proportional bias in all three rings. The BA plot for the Oura Ring indicates significant positive bias (overestimation) for low levels of SE measurement and significant negative bias (underestimation) for very high measures of SE. For the SleepOn ring, there is significant negative bias across the whole range of measurement, with highest overestimation for low values of reference SE. For Circul, SE measures were significantly overestimated for very low measures of SE and significantly underestimated for high levels of SE.

#### Sleep onset latency

SOL was underestimated, on average, by 3.18 min by the Oura ring (SD = 12.52 min.; error range − 34.50 to 32.50 min.), by 19.11 min by the Sleep On ring (SD = 23.56 min.; error range − 88.00 to 7.00 min.), and by 25.24 min by the Circul ring (SD = 17.82 min.; -83.00 to 0.00 min.). SOL measures of the Oura ring significantly overestimated the reference for very low values of SOL and significantly underestimated the reference for medium and high values of SOL. The SleepOn ring significantly underestimated the reference for all but very low true values of SOL. Significant negative constant bias was present for Circul measurements of SOL, indicating underestimation of SOL values.

#### Wake after sleep onset

On average, WASO reference values were underestimated by -8.56 min by the Oura ring (SD = 33.02 min.; error range from − 90.5 to 66.50 min.) and by 31.34 min by the SleepOn ring (SD = 32.19 min.; error range from − 116.00 to 46.50 min.). The Circul ring overestimated WASO by 56.69 min (SD = 61.62 min.; error range from − 32.00 to 173.00 min.). Oura and SleepOn measurements of WASO were affected by significant proportional bias, with significant underestimation of WASO for medium and high values of WASO. WASO measurements from the Circul ring were affected by significant proportional bias, with significant overestimation of low and medium true values of WASO.

#### Light sleep

Light sleep duration was underestimated by -13.84 min by the Oura ring (SD = 79.43 min.; error range from − 162.00 to 234.50 min.), overestimated by 15.99 min by the SleepOn ring (SD = 67.67 min.; error range from − 112.50 to 166.00 min.), and underestimated by 18.55 min by the Circul ring (SD = 63.92 min.; error range from − 153.50 to 136.50 min.), on average. Both the Oura ring and the Circul ring were affected by significant proportional bias. The Oura ring significantly overestimated low values and significantly underestimated high true values of light sleep duration, whereas the Circul ring significantly underestimated high true values of light sleep duration. The SleepOn ring was not affected by significant bias.

#### Deep sleep

Deep sleep duration was underestimated by -5.98 min by the Oura ring (SD = 67.79 min.; error range from − 211.50 to 141.50 min.), overestimated by 31.79 min by the SleepOn ring (SD = 61.33 min.; error range from − 80.00 to 172.50 min.), and underestimated by 15.13 min by the Circul ring (SD = 50.25 min.; error range from − 122.00 to 63.00 min.). Similarly to light sleep duration, deep sleep duration measurements also exhibited biases: Both the Oura and the Circul ring were affected by significant proportional bias, leading to significant overestimation for low true values of deep sleep duration and significant underestimation of medium and high true values of deep sleep duration. Significant constant bias indicating device overestimation was present for the SleepOn ring.

#### REM sleep

REM sleep duration was overestimated by 31.56 min by the Oura ring (SD = 44.04 min.; error range from − 40.00 to 172.00), by 2.69 min by the SleepOn ring (SD = 57.40 min.; error range from − 67.50 to 153.00 min.), and by 2.23 min by the Circul ring (SD = 37.86 min.; error range from − 111.00 to 67.50 min.). For the Oura ring, significant, positive constant bias was present, indicating device overestimation. No significant bias was detected for the SleepOn ring. The Circul ring was affected by significant proportional bias, leading to significant device overestimation for low true values of REM sleep duration and significant device underestimation for high true values of REM sleep duration.

### Epoch-by-epoch analysis

Tables 3 and 4 report the epoch-by-epoch group-level proportional error matrix for each ring for sleep state classification (i.e., binary sleep/wake distinction) and sleep stage classification (i.e., four-label distinction between wake, light, deep, and REM sleep), respectively. First, individual-error matrices were computed for each participant, and each cell value was divided by its corresponding marginal reference value, resulting in the average proportion of each class over the number of epochs classified as the corresponding class by the reference. Average values across subjects were then computed. Standard deviations indicate the variability in agreement and error around the mean while confidence intervals indicate the precision of the mean. Thus, values on the diagonal of each confusion matrix indicate sensitivity, i.e., the ability of the device to correctly classify true labels (e.g., 46% of epochs classified as Wake by PSG were classified as Wake by the Oura ring; SD = 19%; Table 4). Non-diagonal column entries indicate classification errors for each true label (e.g., true wake epochs that were erroneously classified as light, sleep, or REM, in the first column of Table 4).

In binary sleep/wake distinction, all three rings exhibit a typical pattern, in that sensitivity to detect sleep is higher than sensitivity to detect wake (Table 3), which is an expected pattern given that typically, most of the night is spent sleeping rather than awake, leading to a class imbalance. Oura and SleepOn exhibited similar overall accuracy, whereas the Circul ring performed worse (Oura: 85.03%, Kappa = 0.43; SleepOn: 65%, Kappa = 0.44; Circul: 65.66%, Kappa = 0.10). As has been found in prior sleep measurement performance analyses, high accuracy values can hide the fact that the less frequent wake epochs are not accurately detected, as shown by much lower wake sensitivity / sleep specificity and Kappa values. During sleep stage classification, all three rings frequently misclassified true wake epochs as light sleep. In particular, the SleepOn ring more frequently classified wake epochs as light sleep than as wake. The reverse is not necessarily true, as only the Circul ring frequently misclassified light sleep as wake, whereas the Oura and the SleepOn ring rather tend towards misclassifications as REM sleep or Deep sleep. Epochs classified as deep sleep by PSG were frequently classified as Light sleep by all three rings. This is particularly striking for the Circul ring, which correctly sensed only 17.00% of deep sleep epochs (SD = 18%; Kappa = 0.03), while labeling 52.00% of deep sleep epochs as light sleep (SD = 24%). Similarly, REM sleep classification of the Circul ring performed only at 14.00% sensitivity (SD = 14%; Kappa = 0.01) as the ring often misclassified REM sleep epochs as wake of light sleep. Aggregating over all four sleep stages, the Oura ring achieved 53.18% accuracy (Kappa = 0.31, SE = 0.00, CI = [0.31, 0.32]), the SleepOn ring performed at 50.48% accuracy (Kappa = 0.27, SE = 0.00, CI = [0.27, 0.28]), and the Circul ring achieved 35.06% accuracy (Kappa = 0.06, SE = 0.00, CI = [0.05, 0.06]).

### Questionnaire responses

**Fig. 4:**
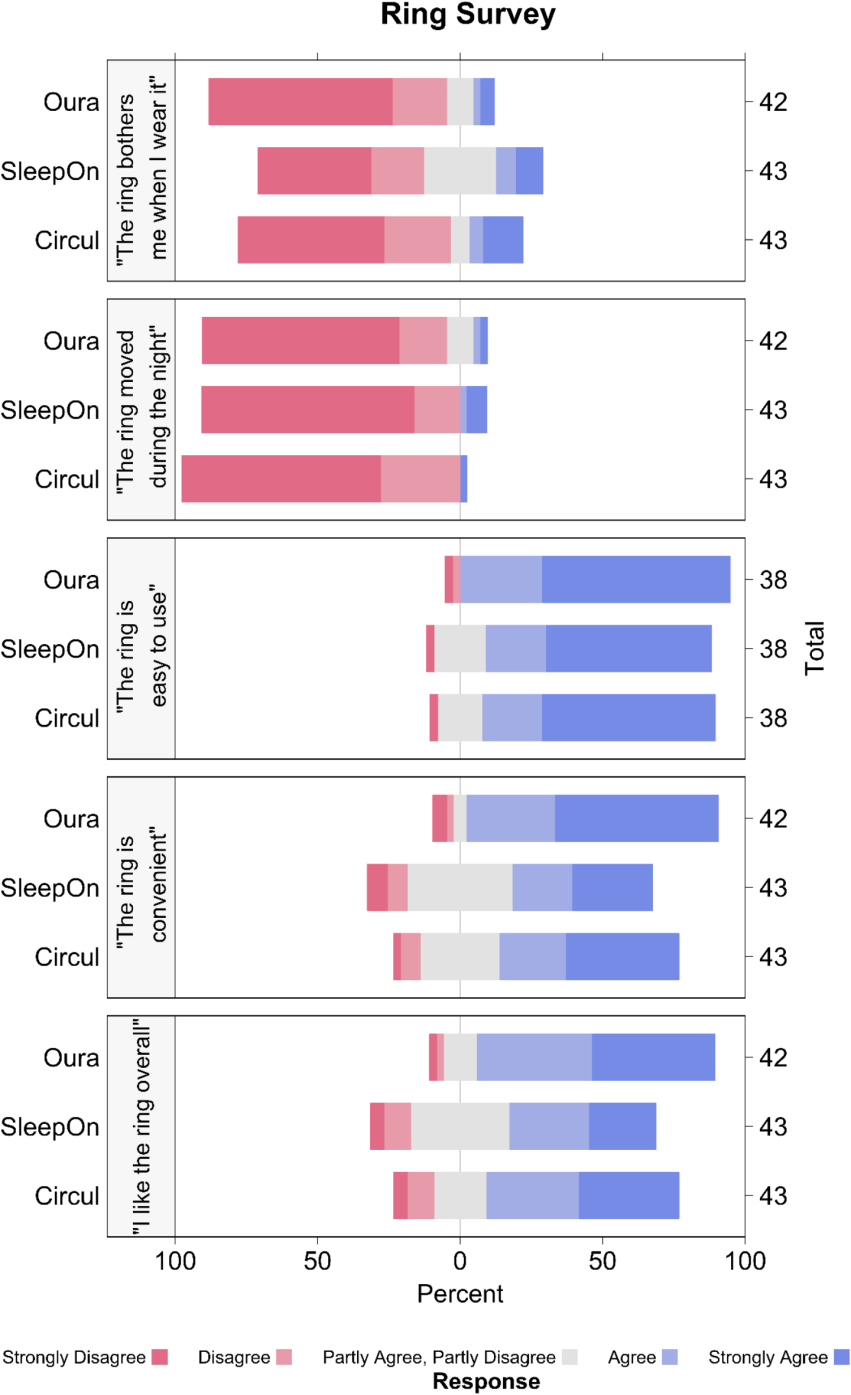
Responses to the questionnaire for all five questions and for each ring. Likert scale responses are represented as percentage relative to total response counts (indicated on the right y-axis).

Forty-two participants reported for the Oura ring, whereas both the SleepOn and the Circul ring were rated by 43 participants (Fig. 4). Four participants indicated they could not answer the third question (“The ring is easy to use”). Overall, responses were positive, with most participants responding that they disagreed or strongly disagreed with the statement that the ring bothers them and the statement that the ring moved during the night. Similarly, most participants agreed or strongly agreed that the rings were easy to use, are convenient, and that they liked the ring overall. The SleepOn ring tracker, which has a prominent, oval shaped compartment attached to the ring, was rated lower for question 1 (“The ring bothers me when I wear it”) and question 4 (“The ring is convenient”).

## Discussion

All three rings were generally rated favorably in comfort and ease of use by the participants. However, we did experience significant data dropouts resulting from ring recordings errors or poor fit, highlighting the possibility of data loss with user-friendly ring tracker devices.

Assessing sleep metrics (TST, SE, SOL, WASO, Light, Deep, REM sleep duration) in our sample of sleep lab patients with a wide set of clinically relevant conditions, we observe that while ring trackers are reasonably aligned with the gold standard (PSG) *on average*, large variation in errors at the individual-level are apparent. Further, when examining group-level and individual-level discrepancies between sleep tracking devices and a gold-standard reference on sleep metrics, we found that device-reference differences were often not affected by strictly constant bias – which could easily be corrected by subtracting a calibration index (i.e., the average difference across participants) – but rather were influenced by proportional bias, meaning that correction of device measurements would have to be adjusted based on the scale of measurement (see similar findings by Chinoy and colleagues^17^ for seven other sleep tracking devices). Limits of agreement computed for each sleep measure and for each ring highlighted that for some sleep measurements, large deviations between sleep tracking devices and ring trackers should be expected. For instance, limits of agreement between device and reference measures indicate that 95% of expected difference values for light sleep duration would fall in between roughly 100 min above and below the bias line. Further, for several sleep measures, individual participants fell outside of these limits of agreement for specific rings (Figs. 2, 3 and 4). In sum, while it may be possible to account for systematic biases between ring tracker devices and reference, individual differences between ring tracker and PSG measures are too large for any professional use in a clinical or diagnostic context.

In contrast to previous works that concluded that sleep/wake classification can be achieved with consumer grade equipment at reasonable accuracy despite medium sensitivity^17–19^, our results from a sleep lab sample reinforce that high accuracy values are often overshadowed by low wake state sensitivity (Oura: 46.19, SleepOn: 37.40, Circul: 35.43). Additionally, our results support the finding that poor/disrupted sleep can decrease accuracy^17^. Wake periods are difficult to classify correctly in data dominated by a majority of sleep epochs, often leading to large measurement discrepancies. However, accurate detection of wake state is critical for the correct assessment of the quantitative sleep measures (TST, SE, SOL, WASO) in clinical sleep medicine and scientific domains. Going beyond the binary distinction of epochs of sleep and wake state, the correct classification of sleep stage is a strong requirement for clinical and scientific measurement of sleep, as performed in PSG. Overall, our results suggest very limited accuracy of sleep staging into the classes wake, light sleep, deep sleep, and REM sleep, with the Oura ring achieving the best accuracy (53.18%), followed closely by the SleepOn ring (50.48%), whereas the Circul ring performed worst (35.06%). Sleep staging agreement ranged from 58.00% agreement in light sleep detection by the SleepOn ring to only 14.00% agreement in REM sleep detection by the Circul ring. Interestingly, all three rings often incorrectly labeled wake epochs as light sleep and, similarly, all three rings often incorrectly labeled deep sleep epochs as light sleep. This highlights that ring trackers are currently unable to accurately detect and distinguish between sleep stages. Sensitivity and accuracy were, however, not always following the same patterns for all three rings. For instance, the Oura ring exhibited better performance in classifying REM sleep than the SleepOn and Circul rings. It may thus be possible that the choice of ring depends also on the most important end-point measures for a given sleep study. Interestingly, while the sleep staging labels produced by the ring trackers themselves require improvement, previous work demonstrated better results by training machine learning classifiers on raw device sensor measures against gold-standard labels not only for sleep/wake discrimination^15,16^, but also for sleep staging^16^.

Direct comparisons of our results obtained from sleep-lab patients reveal limited consistency with prior work that was conducted with healthy participants. In case of the Oura ring, previous work found no significant bias for the summary variables TST, SOL, WASO, and light sleep duration^19^. In contrast, our results revealed significant proportional bias for all tested variables, with the exception of REM sleep duration, which was affected by significant constant bias. Similarly, a reported constant bias for deep sleep duration was found to be proportional instead in our results. Interestingly, while this previous work found negative constant bias for REM sleep duration, we instead found positive constant bias, further putting the reliability of ring derived sleep measures across different participant populations into question. Results reported by Chee and colleagues indicated biased recordings of the Oura ring for TST, WASO, and all three sleep stage durations (light, deep, REM) for a sample of adolescent participants^29^, where the aforementioned results do not always overlap. Both studies found good performance in sleep/wake discrimination but, in line with our results, found that sleep staging classification requires improvement (see also convergent results for the 2nd Generation Oura ring^17,18^). Two studies (which declared a conflict of interest with Oura) have reported higher accuracy not only for sleep state but also for sleep stage classification^30,31^. While both studies provide large sample sizes, the sample was selected to include young and healthy individuals and specifically exlcuded participatns with sleep disorders. Our work thus raises the question to what extent the reported performance generalizes to individuals suffering from sleep disorders. Indeed, our results for the sleep characteristics indicate that while ring trackers provide more accurate sleep characteristics in the healthy range (e.g., high values of sleep efficiency), their estimation of clinically relevant values is often not accurate (e.g., low values of sleep efficiency are overestimated by all three rings)^17^. Data on the SleepOn and Circul rings are less abundant. While Chacon and colleagues reported no significant differences between sleep measures and sleep duration for the SleepOn ring compared to reference^32^, we found significant constant bias for deep sleep duration and proportional bias for TST, SE, SOL, and WASO. Zhao and colleagues examined the Circul ring but only focused on blood oxygen related measures^33^ and, thus, no direct comparison to the sleep measures examined here is possible.

We point out several limitations to the current study. The focus of the present work lies on the performance of ring trackers applied directly to the patient, without a longer calibration period, in which the ring can adapt to the individual. Thus it is possible that long-term results from the rings differ from the ones we presented here. Our present work does not encompass analyzing the performance of ring trackers with regard to demographics (age, gender) and specific sleep disorders, both of which are important avenues for future research. The ecological validity of our findings is limited by the parallel application of PSG and three sleep tracker rings at the same time, potentially altering participants’ usual sleeping behavior (see Altini and Kinnunen for discussion^16^). Future work should also be dedicated to investigate the role of ring placement on the right vs. the left hand, as previous work on sleep/wake discrimination based on electrodermal activity revealed lateralization effects^34^. Variability in sleep-related disorders and comorbidities is a feature of the current study, yet further work is needed to examine ring performance for specific disorders, in larger, more homogenous patient samples. Further, our results are based on recordings from a single night per patient, and thus no statement can be made about the reliability of our findings over longer time spans (see problems in long-term reliability reported for another wearable by Baroni et al.^35^). The same holds for the results of our questionnaire, which was completed based on participants impressions after one night of use. Longitudinal studies are required to assess long-term acceptance of ring trackers^36^. Last, our study was designed to test ring performance in-lab, and thus generalization to in-home settings^37^ is limited.

In summary, our findings reveal limited agreement between previous ring tracker evaluations in healthy, young individuals and our sleep-lab patient sample. While group-level average differences for TST, SE, SOL, and WASO compared to PSG were not excessively large, this apparent agreement masks oftentimes large differences at the extreme ends of the measurement scales as well as substantial individual-level differences. We deem these individual discrepancies between ring tracker estimates and PSG values too large for clinical applications requiring high accuracy, particularly in sleep disorders characterized by deviations from average sleep metrics. Averaging across individuals can be misleading, as it does not guarantee accurate measurement of individual nights, which is, however, precisely what clinical sleep medicine relies on for individual patient assessments. The findings about the finger ring trackers performance in sleep state and sleep stage classification resembled previous findings in the literature describing that sleep state classification suffered from low wake-state sensitivity and sleep stage classification performance was very limited overall. We conclude that while ring trackers might hold value for tracking sleep statistics at the group level, their raw output values are insufficient for any individualized diagnostic or scientific applications.

## Method

### Inclusion & ethics

The study was conducted under local ethics board approval (EA1/018/22). All methods were performed in accordance with the relevant guidelines and regulations.

### Sleep trackers

Three sleep trackers were evaluated against polysomnography, which served as the gold standard. The devices used in the investigation were the Oura Ring (size 8 and 12; Gen. 3, Oura Health Oy, Finland)^26^, SleepOn Go2Sleep (SleepOn, USA)^27^, and Circul + Ring (size L and XL; Bodymetrics, USA)^28^. The Oura ring is equipped with sensors providing insight into blood oxygen levels (LED), continuous heart rate (LED), resting heart rate, heart rate variability (HRV) and respiration (PPG), skin temperature (NTC), and movement/activity (accelerometer). While the precise sensors for the SleepOn ring are not made public, the ring provides heart rate, blood oxygen levels and movement data. The Circul + ring measures heart rate and HRV, blood oxygen, ECG, blood pressure, and finger temperature. While the SleepOn ring activates and deactivates when removed from and placed back into its charging port, the Oura ring and the Circul ring are activated and deactivated from in-app instructions. All three rings were updated to their most recent mobile software version. The rings were connected to a standard Apple iPhone (SE, 6) with the latest operating system. Data were exported from the corresponding applications. As the main focus of the present work lies on the epoch-by-epoch sleep staging classification performance of the rings, additional measures recorded by some of the rings (such as heart-rate or SpO2) are not analyzed here. While ring tracker manufacturers advise to wear the rings for prolonged periods of time to ensure calibration of the ring to the individual, we deem this often infeasible in clinical contexts, due to potentially limited amount of rings available and long calibration periods creating delays until treatment can be provided. Due to these reasons, all ring trackers were applied for the first time in the night of data collection.

### Polysomnography

In parallel to the use of the three rings, a standard diagnostic polysomnography (PSG) was performed using the SOMNOtouch device (SOMNOmedics GmbH, Germany). PSG measurements and scoring was carried out by trained professional sleep technician following the latest AASM standard (Version 3)^38^. Recording modalities included EEG, EOG, ECG, EMG, breathing activity, SpO2, as well as sound and video. The PSG EEG montage consisted of six EEG derivations (F3, F4, C3, C4, O1, O2) of the international 10–20 system that were referenced to contralateral mastoids. Following the AASM guidelines, one EOG electrode was placed 1 cm above and one 1 cm below the outer canthus of one eye. During inspection, a 0.3 high pass filter and a 35 Hz low pass filter was applied. While sleep studies should ideally involve multiple scorers^39,40^, in ours case PSG measurements were taken during routine clinical practice and one scorer assigned sleep stage labels for each patient. Patients were randomly assigned to one of two scorers. Nights with PSG recording errors were excluded from further analysis (Suppl. Table 5).

### Participants

All participants gave written and informed consent to their participation in the study. Data were acquired from 45 participants (20 female) aged 23 to 74 years (mean age 54.60 years; SD = 12.20). Participants had an average BMI of 33.50 (SD = 7.70, range 19.60–51.90). Exclusion criteria were drug or alcohol abuse, acute or chronic diseases requiring treatment outside of the scope of sleep medicine, clinically unstable respiratory tract diseases or heart diseases, and participation in clinical pharmacological examinations within four weeks prior to the study. Prior medical sleep diagnoses included light to severe obstructive sleep apnoea syndrome (OSA), severe complex sleep apnoea syndrome (CSA), light to severe mixed sleep apnoea syndrome (MSA), asthma, chronic insomnia, restless-legs-syndrome, hypersomnia, narcolepsy, and nocturnal hypercapnia, as well as other sleep-related and sleep-unrelated disorders (Suppl. Table 2). Participants were not compensated financially for their participation. Demographic data, PSG-derived sleep measures, and apnoea-hypopnoea index (AHI) for each participant are reported in the supplementary section (Suppl. Table 1). Additionally, we provide the sleep disorders diagnosed based on PSG in the clinic (Suppl. Table 2). Due to ring recording errors, not all rings contributed data for each participant. We report averages of demographics (Table 1) and sleep measures from both PSG and each ring tracker for three subsets of participants corresponding to the three ring trackers (Table 2).

### Procedure

Data were acquired in the timeframe of August to November 2022 at Charité University Berlin sleep laboratory in sound attenuated and temperature controlled bedrooms. All participants wore all three rings simultaneously to the PSG measurement on their first night in the sleep lab. Rings were initialized according to their standard protocols and run in parallel to PSG. PSG was applied one to two hours before normal bedtime. Two rings were placed on one hand and one ring was placed on the other, matched to individual fingers according to best individual fit (Oura: right hand 20 times, left hand 24, not applied one time; SleepOn: right hand 28 times, left hand 28, right hand 17 times; Circul: left hand 25 times, right hand 20 times). Sleep technicians confirmed that participants wore the rings properly at bedtime. Before bedtime, and after sensors had been applied, participants engaged in quiet activities. Electronic media usage was discouraged after lights-off, timing which was decided on by participants.

### Questionnaire

Participants completed a survey with 5 questions that were answered for each ring. Responses were indicated on a five-point Likert scale (1: Strongly disagree, 2: disagree 3: Partly agree, partly disagree, 4: Agree, 5: Strongly agree)^41^ investigating user experience, problems and overall satisfaction with the ring. Specifically, the questions were “The ring bothers me when I wear it”, “The ring moved during the night”, “The ring is easy to use”, “The ring is convenient”, in the sense of wieldy, and “I like the ring overall”.

### Data processing

Sleep stages (Awake, REM, N1, N2, N3) were classified based on PSG separately for each participant based on 30 s epochs by expert sleep technicians following the current AASM manual scoring guideline^38^. All three sleep tracker rings provide sleep classification in four classes: wake, light sleep, deep sleep, REM sleep. To align this classification with the standard PSG classification labels, N1 and N2 sleep stages determined by PSG were combined into the label light sleep, and N3 from PSG was considered equivalent to the deep sleep stage, as determined by the ring trackers.

For each tracker, the recording start times with second precision were extracted electronically. Based on this, the sleep staging labels from the three sleep trackers and PSG were aligned using time-stamped epochs. While both PSG and the Circul ring tracker provide sleep stage classifications in 30 s intervals, the SleepOn ring uses 1-minute epochs and the Oura ring uses 5-minute epochs. In order to align these longer intervals with the 30 s intervals used by PSG and Circul, missing observations were filled with the label produced for the entire time span. That is, all 30 s epochs in the 5 min span used by the Oura and the 1 min span of the SleepOn ring were considered to have the same label. In cases where either PSG or the ring tracker produced out of set labels (e.g., “artefact”, “off bed”) or in which data for either one were missing (at the beginning and end of recording), these individual observations were not considered in the calculation of sleep measures and the analysis of agreement between PSG and ring tracker.

Subsequently, several sleep measures were computed from both ring tracker and PSG outputs. While the Oura and SleepOn ring start and stop recording automatically by putting them on, the Circul ring was configured to record between 20:00 and 08:00. Lights-off and Lights-off for the sleep trackers were derived from the PSG recording and only those ring epochs falling within the PSG time range were considered for further analysis. Total sleep time (TST), i.e., the number of minutes classified as sleep between lights-off and lights-on, sleep efficiency (SE), i.e., percentage of TST over time in bed, wake after sleep onset time (WASO), i.e., the number of minutes classified as wake after the first epoch classified as sleep, sleep onset latency (SOL), i.e., the number of minutes classified as wake before the first epoch classified as sleep. Furthermore, in both PSG and ring trackers, TST was computed as the sum of light sleep, deep sleep, and REM sleep stages. SOL (based on which TST was determined) was defined as the first epoch of sleep, regardless of sleep stage. WASO was calculated as the number of minutes classified as wake after the first epoch classified as sleep. SE was determined as the percentage of time in bed that contributed to TST in percent.

### Evaluation

The diagnostic performance of 3 consumer-grade finger ring trackers was compared to in-lab polysomnography (PSG). Evaluation of the three sleep tracker rings was conducted following the standardized framework proposed by Menghini and colleagues^25^ for the comparison of consumer sleep technology to reference PSG. Analysis was conducted using the R reference implementation provided with the framework^42^. The previously described raw data processing steps ensure compatibility of the present data with the analysis framework, resulting in 30 s epoch intervals for all three sleep trackers, aligned with gold standard PSG. In line with the analysis framework, ring trackers and PSG are referred to as “device” and “reference”, respectively. Analysis consists of an individual-level and a group-level discrepancy analysis investigating TST, SE, SOL, SE, as well as duration of light, deep, and REM sleep, as calculated from ring tracker and PSG raw data. Sleep staging classification performance is examined in an epoch-by-epoch error analysis.

### Discrepancy analysis

Bias is assessed quantitatively, by testing for the presence or absence of constant bias (i.e., systematic offset between device and reference measures) or proportional bias (i.e., a systematic offset that increases or decreases as a function of the size of measurement), as proposed in the reference analysis framework^25^. Bias significance in case of constant bias is determined based on whether the zero line falls within the 95% confidence interval or, in case of proportional bias, through the significance of the slope coefficient of the proportional bias regression. In case data deviate from normality, bootstrapped confidence intervals are computed. Visually, bias estimates are represented by red solid lines together with red dashed lines indicating 95% confidence intervals. Estimated biases can be used to adjust the sleep tracker measurements by subtracting the indicated bias amount from them.

Calculation of the limits of agreement (LOAs) is adjusted based on compliance of the data with the assumptions of homoscedasticity and normally distributed differences (see the original framework for full detail). Upper and lower LOA estimates are represented visually by gray lines together with 95% confidence intervals. LOAs indicate thresholds within which 95% of the differences between device and reference may be expected, thereby indicating the degree of measurement agreement between device and reference. Thus, LOAs represent hypothetical thresholds required to accept differences between device and reference measurements.

The computed bias and LOA terms inform both individual-level and group-level discrepancy analyses. Discrepancies are assessed at the group-level by reporting device and reference averages for the aforementioned sleep measures, as well as the aforementioned estimates for device bias and limits of agreement (LOA), both supported by confidence intervals. In addition to the group-level analysis, individual-level discrepancies are examined visually through Bland-Altman plots^43^. For each sleep measure, the difference between device and reference measures for each participant are plotted against the average reference value for each participant, allowing to visually assess systematic over- and underestimation of device estimates, relative to the entire range reference values.

### Epoch-by-epoch analysis

Focusing on sleep staging classification performance, a group-level epoch-by-epoch (EBE) analysis is conducted, resulting in an error matrix which informs about the proportion of classification agreement and classification error between device and reference, for each of the four sleep stages. Further, we report overall accuracy in sleep stage classification accuracy. Overall sleep state detection accuracy is computed from sleep staging classifications, by merging light, deep, and REM sleep into a single “sleep” class post-hoc.

## Electronic supplementary material

Below is the link to the electronic supplementary material.

Supplementary Material 1

## Data Availability

All data analyzed in this study and all code necessary for reproducing the results are available upon reasonable request by inquiry to the corresponding author Sebastian Herberger.
