# Supplementary material for: Performance of wearable finger ring trackers for diagnostic sleep measurement in the clinical context
Source: Sci Rep. 2025 Mar 19;15:9461. doi: 10.1038/s41598-025-93774-z (PMC11923143; doi:10.1038/s41598-025-93774-z)
Supplement: Supplementary file 1 — Supplementary Material 1 [file 41598_2025_93774_MOESM1_ESM.docx]

# Supplementary Information: Tables

| Sub. | Age | Gen. | Ring Size (M-L) | PSG | Oura | SleepOn | Circul | TIB | TST (m) | SE (%) | SOL (m) | WASO (m) | Light (m) | Deep (m) | REM (m) | Light (%) | Deep (%) | REM (%) | AHI |
| --- | --- | --- | --- | --- | --- | --- | --- | --- | --- | --- | --- | --- | --- | --- | --- | --- | --- | --- | --- |
| 1 | 70 | m | M | 1 | 1 | 1 | 1 | 448.5 | 383 | 85.4 | 9 | 56.5 | 294.5 | 36 | 52.5 | 76.89 | 9.4 | 13.71 | 57.5 |
| 2 | 74 | m | L | 0 | 0 | 0 | 0 |  |  |  |  |  |  |  |  |  |  |  |  |
| 3 | 60 | f | L | 1 | 1 | 1 | 1 | 224.5 | 128.5 | 57.24 | 34.5 | 61.5 | 105 | 1.5 | 22 | 81.71 | 1.17 | 17.12 | 77.5 |
| 4 | 55 | f | M | 0 | 0 | 0 | 0 |  |  |  |  |  |  |  |  |  |  |  |  |
| 5 | 61 | f | M | 1 | 0 | 0 | 1 | 426.5 | 293 | 68.7 | 22.5 | 111 | 170 | 81 | 42 | 58.02 | 27.65 | 14.33 | 9.4 |
| 6 | 56 | m | L | 1 | 1 | 1 | 1 | 547 | 437 | 79.89 | 52 | 58 | 361 | 47 | 29 | 82.61 | 10.76 | 6.64 | 23.3 |
| 7 | 55 | f | M | 1 | 1 | 1 | 1 | 420 | 345.5 | 82.26 | 12 | 62.5 | 140.5 | 119.5 | 85.5 | 40.67 | 34.59 | 24.75 | 9 |
| 8 | 57 | m | L | 1 | 1 | 1 | 0 | 532 | 411.5 | 77.35 | 94.5 | 26 | 194 | 108 | 109.5 | 47.14 | 26.25 | 26.61 | 42 |
| 9 | 49 | m | M | 1 | 0 | 1 | 1 | 539 | 381 | 70.69 | 93.5 | 64.5 | 304.5 | 26 | 50.5 | 79.92 | 6.82 | 13.25 | 80.3 |
| 10 | 48 | m | M | 1 | 0 | 1 | 1 | 513 | 447 | 87.13 | 42 | 24 | 381.5 | 14 | 51.5 | 85.35 | 3.13 | 11.52 | 74.4 |
| 11 | 31 | m | L | 1 | 1 | 1 | 0 | 448 | 398.5 | 88.95 | 9.5 | 40 | 236 | 85.5 | 77 | 59.22 | 21.46 | 19.32 | 10.2 |
| 12 | 65 | f | M | 1 | 1 | 1 | 1 | 540 | 454 | 84.07 | 33.5 | 52.5 | 185.5 | 211.5 | 57 | 40.86 | 46.59 | 12.56 | 9.4 |
| 13 | 58 | f | M | 1 | 1 | 1 | 1 | 508.5 | 365 | 71.78 | 52.5 | 91 | 254 | 58.5 | 52.5 | 69.59 | 16.03 | 14.38 | 40.9 |
| 14 | 66 | m | L | 1 | 1 | 1 | 1 | 464 | 391.5 | 84.38 | 20.5 | 52 | 225.5 | 109 | 57 | 57.6 | 27.84 | 14.56 | 8 |
| 15 | 61 | f | M | 1 | 1 | 1 | 1 | 445 | 327.5 | 73.6 | 43 | 74.5 | 225 | 57 | 45.5 | 68.7 | 17.4 | 13.89 | 17.1 |
| 16 | 60 | m | L | 0 | 0 | 0 | 0 |  |  |  |  |  |  |  |  |  |  |  |  |
| 17 | 49 | m | L | 1 | 1 | 0 | 1 | 466.5 | 393 | 84.24 | 18 | 55.5 | 283.5 | 14.5 | 95 | 72.14 | 3.69 | 24.17 | 9.6 |
| 18 | 52 | f | M | 1 | 0 | 1 | 1 | 425.5 | 382.5 | 89.89 | 6 | 37 | 191.5 | 5.5 | 185.5 | 50.07 | 1.44 | 48.5 | 20.2 |
| 19 | 30 | f | L | 0 | 0 | 0 | 0 |  |  |  |  |  |  |  |  |  |  |  |  |
| 20 | 60 | m | L | 0 | 0 | 0 | 0 |  |  |  |  |  |  |  |  |  |  |  |  |
| 21 | 59 | f | M | 1 | 1 | 1 | 1 | 521.5 | 324 | 62.13 | 85.5 | 112 | 199.5 | 93 | 31.5 | 61.57 | 28.7 | 9.72 | 36.9 |
| 22 | 69 | f | L | 1 | 1 | 1 | 0 | 535 | 279 | 52.15 | 161 | 95 | 135 | 129 | 15 | 48.39 | 46.24 | 5.38 | 8.2 |
| 23 | 60 | m | L | 1 | 1 | 1 | 1 | 386 | 332.5 | 86.14 | 30 | 23.5 | 189.5 | 83.5 | 59.5 | 56.99 | 25.11 | 17.89 | 37.7 |
| 24 | 27 | m | M | 1 | 0 | 0 | 0 | 540 | 495.5 | 91.76 | 37.5 | 7 | 174.5 | 162 | 159 | 35.22 | 32.69 | 32.09 | 5.2 |
| 25 | 63 | m | L | 1 | 1 | 1 | 1 | 498.5 | 364 | 73.02 | 50 | 84.5 | 248 | 50.5 | 65.5 | 68.13 | 13.87 | 17.99 | 22.9 |
| 26 | 68 | f | M | 1 | 1 | 1 | 1 | 470.5 | 292.5 | 62.17 | 38 | 140 | 163.5 | 79.5 | 49.5 | 55.9 | 27.18 | 16.92 | 6.6 |
| 27 | 56 | m | L | 1 | 1 | 1 | 1 | 635.5 | 487.5 | 76.71 | 135.5 | 12.5 | 215.5 | 158 | 114 | 44.21 | 32.41 | 23.38 | 20.2 |
| 28 | 55 | m | L | 1 | 1 | 1 | 0 | 430 | 389.5 | 90.58 | 9 | 31.5 | 200 | 105 | 84.5 | 51.35 | 26.96 | 21.69 | 13.1 |
| 29 | 62 | f | M | 1 | 0 | 1 | 0 | 502.5 | 280 | 55.72 | 71 | 151.5 | 217 | 37.5 | 25.5 | 77.5 | 13.39 | 9.11 | 69.4 |
| 30 | 68 | f | L | 1 | 1 | 0 | 1 | 397.5 | 257 | 64.65 | 14 | 126.5 | 178 | 61.5 | 17.5 | 69.26 | 23.93 | 6.81 | 23.3 |
| 31 | 58 | m | M | 1 | 1 | 1 | 1 | 432.5 | 354.5 | 81.97 | 9 | 69 | 152.5 | 153.5 | 48.5 | 43.02 | 43.3 | 13.68 | 7.5 |
| 32 | 60 | f | M | 1 | 1 | 1 | 1 | 547.5 | 372 | 67.95 | 40 | 135.5 | 238 | 53.5 | 80.5 | 63.98 | 14.38 | 21.64 | 48.9 |
| 33 | 48 | m | L | 1 | 1 | 0 | 1 | 463.5 | 416 | 89.75 | 4 | 43.5 | 291 | 32 | 93 | 69.95 | 7.69 | 22.36 | 79.8 |
| 34 | 23 | m | M | 1 | 1 | 1 | 0 | 487 | 408 | 83.78 | 18 | 61 | 269 | 60 | 79 | 65.93 | 14.71 | 19.36 | 1.2 |
| 35 | 40 | m | L | 1 | 1 | 1 | 0 | 450.5 | 358.5 | 79.58 | 21 | 71 | 224.5 | 37.5 | 96.5 | 62.62 | 10.46 | 26.92 | 40.3 |
| 36 | 68 | f | M | 1 | 0 | 1 | 1 | 470 | 376 | 80 | 39 | 55 | 248 | 116 | 12 | 65.96 | 30.85 | 3.19 | 33 |
| 37 | 57 | m | L | 1 | 0 | 1 | 1 | 434 | 305.5 | 70.39 | 27 | 101.5 | 108 | 118.5 | 79 | 35.35 | 38.79 | 25.86 | 24.4 |
| 38 | 67 | m | L | 1 | 0 | 1 | 0 | 435 | 190 | 43.68 | 62 | 183 | 109.5 | 72 | 8.5 | 57.63 | 37.89 | 4.47 | 2.5 |
| 39 | 53 | f | M | 1 | 1 | 1 | 1 | 569.5 | 524.5 | 92.1 | 17 | 28 | 284.5 | 147.5 | 92.5 | 54.24 | 28.12 | 17.64 | 11.3 |
| 40 | 36 | m | L | 1 | 1 | 1 | 0 | 431.5 | 391 | 90.61 | 19 | 21.5 | 219.5 | 107.5 | 64 | 56.14 | 27.49 | 16.37 | 2.9 |
| 41 | 64 | f | M | 1 | 1 | 1 | 1 | 460 | 417.5 | 90.76 | 15.5 | 27 | 283.5 | 49.5 | 84.5 | 67.9 | 11.86 | 20.24 | 8 |
| 42 | 31 | m | L | 1 | 1 | 1 | 1 | 451.5 | 332.5 | 73.64 | 32.5 | 86.5 | 195 | 80.5 | 57 | 58.65 | 24.21 | 17.14 | 4.7 |
| 43 | 46 | m | M | 1 | 1 | 0 | 1 | 456 | 354 | 77.63 | 31.5 | 70.5 | 201.5 | 109 | 43.5 | 56.92 | 30.79 | 12.29 | 13.1 |
| 44 | 50 | f | L | 1 | 1 | 1 | 1 | 465.5 | 388 | 83.35 | 6 | 71.5 | 238 | 71 | 79 | 61.34 | 18.3 | 20.36 | 7.9 |
| 45 | 54 | f | M | 1 | 0 | 1 | 1 | 446.5 | 375 | 83.99 | 0 | 71.5 | 226.5 | 69.5 | 79 | 60.4 | 18.53 | 21.07 | 21.4 |

Suppl. Table 1: Individual-level demographics and sleep measures for each subject. No sleep measures are available for subjects where PSG data were not acquired successfully.

| Sub. | Diagnosis |
| --- | --- |
| 1 | Severe mixed SAS, Epilepsy |
| 2 | Severe OSAS, RLS |
| 3 | Severe OSAS |
| 4 | Preeminently hypersomnia |
| 5 | Severe OSAS, PLMD |
| 6 | Severe OSAS |
| 7 | Severe complex sleep apnea syndrome, PLMD |
| 8 | Severe complex sleep apnea syndrome |
| 9 | OSAS |
| 10 | Severe OSAS, Suspected OHS, Respiratory Insufficiency |
| 11 | Severe, mixed, predominantly obstructive SAS, Suspected Obesity Hypoventilation Syndrome |
| 12 | Severe Insomnia |
| 13 | Severe OSAS overlapping with COPD and Asthma |
| 14 | Severe sleep-related breathing disorder with periodic breathing |
| 15 | OSAS, other sleep disorder |
| 16 | OSAS |
| 17 | Complex sleep apnea, Hypersomnia (possibly residual daytime sleepiness under PAP therapy (differential diagnosis Narcolepsy)), Suspected REM sleep behavior disorder |
| 18 | Narcolepsy Type 1, OSAS, Suspected RBD, Chronic Insomnia, RLS |
| 19 | Severe OSAS |
| 20 | Severe OSAS, Insomnia, Suspected RLS, Suspected PLMD |
| 21 | Severe mixed sleep apnea syndrome, RLS |
| 22 | OSAS |
| 23 | Severe OSAS |
| 24 | Suspected Hypersomnia in Depression, Suspected Inadequate Sleep Hygiene Syndrome |
| 25 | OSAS |
| 26 | Mild OSAS |
| 27 | Severe mixed sleep apnea syndrome, Suspected Hypersomnia DD Narcolepsy Type 2 DD EDS due to sleep-related breathing disorder, PLMS |
| 28 | OSAS |
| 29 | OHS DD severe OSAS, Suspected RLS |
| 30 | OSAS, chronic insomnia, RLS |
| 31 | Severe OSAS |
| 32 | Severe OSAS |
| 33 | Severe OSAS |
| 34 | Mild OSAS |
| 35 | Severe OSAS |
| 36 | Central periodic breathing disorder, intermittent nocturnal hypercapnia |
| 37 | Pavor nocturnus/Night terrors (not breathing related, from N2 sleep), moderate sleep apnea, problems falling asleep and staying asleep, nightmares, unrefreshing sleep, daytime sleepiness, difficulty concentrating |
| 38 | Moderate OSAS, severe chronic insomnia, RLS |
| 39 | Mild OSAS, hypoxic respiratory insufficiency |
| 40 | Mild mixed sleep apnea syndrome, Suspected Parasomnia, Bruxism (teeth grinding) |
| 41 | Nocturnal hypercapnia in mitochondrial myopathy |
| 42 | Suspected Parasomnia |
| 43 | Suspected Hypersomnia DD Narcolepsy, RLS |
| 44 | Severe OSAS |
| 45 | Moderate to severe OSAS (supine position-associated) |

Suppl. Table 2: Sleep disorders diagnosed based on PSG.

|  | **Measure** | **Device Mean (SD)** | **Reference Mean (SD)** | **Bias [95% CI]** | **LOAs [95% CI]** | **Upper LOA [95% CI]** |
| --- | --- | --- | --- | --- | --- | --- |
| **Oura** | TST (min) | 379.11 (70.49) | 367.37 (74.67) | 77.12 + -0.18 x ref  b0 = [11.09, 143.15],  b1 = [-0.35, 0]* | bias - 67.9  bias - [54.53, 87.49] | bias + 67.92  bias + [54.53, 87.49] |
|  | SE (%) | 86.42 (7.65) | 83.4 (9.55) | 61.71 + -0.7 x ref  b0 = [41.15, 104.52],  b1 = [-1.19, -0.46]* | bias - reference x 0.25  bias - reference x [0.18, 0.35] | bias + reference x 0.25  bias + reference x [0.18, 0.35] |
|  | SOL (min) | 6.44 (7.42) | 9.61 (8.51) | 8.37 + -1.2 x ref  b0 = [4.26, 12.47],  b1 = [-1.52, -0.88]* | bias - 14.15  bias - [8.67, 21.04] | bias + 14.15  bias + [8.67, 21.04] |
|  | WASO (min) | 51.98 (29.94) | 60.55 (34.97) | 26.51 + -0.58 x ref  b0 = [6.78, 46.23],  b1 = [-0.86, -0.3]* | bias - 51.11  bias - [38.28, 69.79] | bias + 51.11  bias + [38.28, 69.79] |
|  | Light sleep (min) | 200.16 (69.85) | 214 (47.27) | 161.66 + -0.82 x ref  b0 = [39.68, 283.64],  b1 = [-1.38, -0.26]* | bias - 135.89  bias - [95.3, 183.8] | bias + 135.89  bias + [95.3, 183.8] |
|  | Deep sleep (min) | 79.76 (56.59) | 85.74 (48.35) | 62.43 + -0.8 x ref  b0 = [19.5, 105.37],  b1 = [-1.24, -0.36]* | bias - 109.26  bias - [85.44, 147.24] | bias + 109.26  bias + [85.44, 147.24] |
|  | REM (min) | 99.19 (51.5) | 67.63 (31.26) | 31.56 (44.04)  [15.61, 46.05]* | bias - reference x 0.92  bias - reference x [0.74, 1.18] | bias + reference x 0.92  bias + reference x [0.74, 1.18] |
| **SleepOn** | TST (min) | 418.11 (61.77) | 367.66 (77.54) | 160.45 + -0.3 x ref  b0 = [97.76, 209.7],  b1 = [-0.43, -0.13]* | bias - reference x 0.26  bias - reference x [0.18, 0.36] | bias + reference x 0.26  bias + reference x [0.18, 0.36] |
|  | SE (%) | 89.38 (7.63) | 78.14 (12.28) | 57.03 + -0.59 x ref  b0 = [39.66, 77.45],  b1 = [-0.83, -0.38]* | bias - reference x 0.26  bias - reference x [0.18, 0.36] | bias + reference x 0.26  bias + reference x [0.18, 0.36] |
|  | SOL (min) | 16.33 (24.04) | 35.44 (32.6) | -1.78 + -0.49 x ref  b0 = [-14.89, 6.67],  b1 = [-0.78, -0.02]* | bias - reference x 1.99  bias - reference x [1.98, 2.58] | bias + reference x 1.99  bias + reference x [1.98, 2.58] |
|  | WASO (min) | 34.59 (28.89) | 65.93 (39.13) | 5.96 + -0.57 x ref  b0 = [-10.19, 22.1],  b1 = [-0.78, -0.35]* | bias - 2.46(5.53 + 0.18 x ref)  c0 = [-3.85, 14.91],  c1 = [0.06, 0.3]* | bias + 2.46(5.53 + 0.18 x ref)  c0 = [-3.85, 14.91],  c1 = [0.06, 0.3]* |
|  | Light sleep (min) | 233.63 (78) | 217.64 (63.92) | 15.99 (67.67)  [-7.26, 39.23] | -116.64 [-156.9, -76.38] | 148.61 [108.35, 188.87] |
|  | Deep sleep (min) | 114.97 (75.93) | 83.19 (48.35) | 31.79 (61.33)  [10.72, 52.85]* | -88.42 [-124.9, -51.93] | 151.99 [115.5, 188.48] |
|  | REM (min) | 69.51 (61.98) | 66.83 (37.52) | 2.69 (57.4)  [-16.48, 20.66] | bias - reference x 2  bias - reference x [2, 2.03] | bias + reference x 2  bias + reference x [2, 2.03] |
| **Circul** | TST (min) | 337.73 (88.45) | 369.18 (75.69) | -31.45 (70.06)  [-57.15, -5.75]* | -168.77 [-213.27, -124.26] | 105.86 [61.35, 150.37] |
|  | SE (%) | 71.66 (12.14) | 78.51 (9.67) | 75.21 + -1.05 x ref  b0 = [37.5, 112.93],  b1 = [-1.52, -0.57]* | bias - 23.79  bias - [19.87, 29.88] | bias + 23.79  bias + [19.87, 29.88] |
|  | SOL (min) | 7.29 (25.93) | 32.53 (28.69) | -25.24 (17.82)  [-31.15, -18.79]* | bias - reference x 2  bias - reference x [2, 2.01] | bias + reference x 2  bias + reference x [2, 2.01] |
|  | WASO (min) | 122.89 (59.02) | 66.19 (34.5) | 98.52 + -0.63 x ref  b0 = [54.58, 145.39],  b1 = [-1.24, -0.14]* | bias - reference x 1.36  bias - reference x [1.16, 1.65] | bias + reference x 1.36  bias + reference x [1.16, 1.65] |
|  | Light sleep (min) | 204.35 (70.15) | 222.9 (64.04) | 70.2 + -0.4 x ref  b0 = [-10.32, 150.72],  b1 = [-0.75, -0.05]* | bias - 114.88  bias - [92.42, 147.25] | bias + 114.88  bias + [92.42, 147.25] |
|  | Deep sleep (min) | 64.35 (34.38) | 79.48 (51.28) | 44.91 + -0.76 x ref  b0 = [22.6, 67.23],  b1 = [-0.99, -0.52]* | bias - 62.73  bias - [54.07, 76.24] | bias + 62.73  bias + [54.07, 76.24] |
|  | REM (min) | 69.02 (20.06) | 66.79 (37.36) | 60.29 + -0.87 x ref  b0 = [45.21, 75.37],  b1 = [-1.07, -0.67]* | bias - 2.46(23.73 + -0.12 x ref)  c0 = [15.95, 31.51],  c1 = [-0.22, -0.02]* | bias + 2.46(23.73 + -0.12 x ref)  c0 = [15.95, 31.51],  c1 = [-0.22, -0.02]* |

Suppl. Table 3. Group-level discrepancies of each ring and relevant measurement parameters. TST, total sleep time; SE, sleep efficiency; SOL, sleep onset latency; WASO, wake after sleep onset; Light sleep, (PSG-derived N1 + N2); Deep sleep (PSG-derived N3); REM, rapid eye movement sleep; SD, standard deviation; CI, confidence intervals; LOA, limit of agreement; ref, reference-derived measures (i.e. PSG, used to quantify the size of measurement).

|  | **Oura** | **SleepOn** | **Circul** |
| --- | --- | --- | --- |
| **Wake** | 0.40 (0.18) [0.34, 0.46] | 0.40 (0.18) [0.34, 0.46] | 0.09 (0.26) [0.00, 0.18] |
| **Light** | 0.21 (0.17) [0.15, 0.27] | 0.16 (0.15) [0.11, 0.21] | 0.03 (0.19) [-0.04, 0.10] |
| **Deep** | 0.36 (0.24) [0.28, 0.45] | 0.24 (0.18) [0.18, 0.30] | 0.03 (0.16) [-0.03, 0.08] |
| **REM** | 0.32 (0.28) [0.22, 0.42] | 0.13 (0.19) [0.07, 0.19] | 0.01 (0.16) [-0.05, 0.06] |

Suppl. Table 4. Cohen’s Kappa computed for each ring and in each of the four sleep stages. Results are reported as *mean (standard deviation) [95% confidence intervals]*.

|  | Oura | SleepOn | Circul |
| --- | --- | --- | --- |
| Ring recording error | 6 (13.33%) | 4 (8.89%) | 8 (17.78%) |
| PSG recording error | 1 (2.22%) | 1 (2.22%) | 1 (2.22%) |
| Protocol error | 2 (4.44%) | 2 (4.44%) | 2 (4.44%) |
| Problems with ring fit | 5 (11.11%) | 3 (6.67%) | 3 (6.67%) |
| Total | 14 (31.11%) | 10 (22.22%) | 14 (31.11%) |

Suppl. Table 5. Recording dropout type and rate for each ring tracker.
